# Retraction: A tree of life based on ninety-eight expressed genes conserved across diverse eukaryotic species

**DOI:** 10.1371/journal.pone.0197602

**Published:** 2018-05-14

**Authors:** The PLOS ONE Editors

Following publication of the article, readers raised a number of concerns about aspects of this work, particularly those relating to the phylogenetic tree and the divergence times based on synonymous substitution rates. The *PLOS ONE* Editors have consulted with two members of the Editorial Board who have conducted an independent re-evaluation of the paper, which found concerns regarding the study design, methodology, and interpretation of the data, such that the results of the study were determined to be unreliable. Issues include:

- The findings contradict a large body of existing literature and do not provide a sufficient level of evidence to support the claims made in the paper.
- The selection of *Chlamydomonas* as the outgroup, contrary to the established understanding of evolutionary relationships between green algae, plants, animals, and fungi
- A conceptual flaw in placing *Chlamydomonas* as an outgroup then interpreting the resulting tree as evidence for the basal position of this taxon
- The molecular clock analysis methodology produced a number of inferred divergence times that contradict the fossil record data and also the phylogeny presented in the paper.
- Incorrect interpretation of distance between taxa based on adjacent position in the graphic presentation of the tree

In light of the concerns raised, the *PLOS ONE* Editors retract this article.

PKJ, AS, and NKS agree with the retraction. VD and TRS could not be reached.
